# The value of histopathology combined with CapitalBio *Mycobacterium* real-time polymerase chain reaction test for diagnosing spinal tuberculosis

**DOI:** 10.3389/fmed.2023.1173368

**Published:** 2023-06-23

**Authors:** Liwei Yao, Caifang Bu, Jinjuan Zhang, Dandan Zhang

**Affiliations:** ^1^Department of Nursing, Affiliated Hangzhou Chest Hospital, Zhejiang University School of Medicine, Hangzhou Red Cross Hospital, Hangzhou, Zhejiang, China; ^2^Department of Orthopaedics, Affiliated Hangzhou Chest Hospital, Zhejiang University School of Medicine, Hangzhou Red Cross Hospital, Hangzhou, Zhejiang, China

**Keywords:** histopathology, CapitalBio test, molecular test, spinal tuberculosis, accuracy

## Abstract

**Purpose:**

To evaluate the diagnostic efficacy of CapitalBio *Mycobacterium* real-time polymerase chain reaction assay (CapitalBio test) in spinal tuberculosis (STB). The value of histopathology combined with the CapitalBio test in diagnosing STB was also assessed.

**Methods:**

We retrospectively analyzed the medical information of suspected STB. The sensitivity, specificity, positive predictive value (PPV), negative predictive value (NPV), and area under the curve (AUC) of histopathology, CapitalBio test, and histopathology combined with CapitalBio test were calculated to evaluate their diagnostic efficacy compared with a composite reference standard.

**Results:**

A total of 222 suspected STB patients were included in the study. The sensitivity, specificity, PPV, NPV, and AUC of histopathology for STB were recorded to be 62.0, 98.0, 97.4, 68.3%, and 0.80, respectively. The sensitivity, specificity, PPV, NPV, and AUC of the CapitalBio test were 75.2, 98.0, 97.9, 76.7%, and 0.87, respectively, while that of histopathology combined with the CapitalBio test was 81.0, 96.0, 96.1, 80.8%, and 0.89, respectively.

**Conclusion:**

Histopathology and CapitalBio test exhibited high accuracy and are recommended in diagnosing STB. Histopathology combined with the CapitalBio test might give the best efficacy in STB diagnosis.

## Introduction

Tuberculosis (TB) is an ancient infectious disease that still threatens public health and is caused by *Mycobacterium tuberculosis* (MTB) (1). TB can be classified into pulmonary TB (PTB) and extrapulmonary TB (EPTB), depending on whether the MTB infection affects the lungs (2). EPTB refers to TB of many organs, bones and joints called osteoarticular TB, the more common form of EPTB (3). Approximately 50% of osteoarticular TB is spinal TB (STB), the most common type of osteoarticular TB (4). The spine is a key part of the body’s locomotor system, and within the spine is the spinal cord. Diseases in the spine decrease the system’s stability and affect motor and neurological function (5). The depth of the STB lesion makes it easy to confuse it with other spinal conditions and makes diagnosis difficult. STB tends to destroy the spinal vertebrae, leading to spinal deformity and paraplegia, which seriously affects the patient’s quality of life and the prognosis of the disease (6). Most STB that affects the spine’s stability require surgical treatment to re-establish the integrity and function of the spine. Timely and accurate diagnosis is foundational to reducing treatment delays and improving patient outcomes. Acidfast bacilli (AFB) smear and culture, the standard TB diagnostic procedures exhibit certain limitations, such as the low sensitivity inability to differentiate between TB and nontuberculoaus mycobacteria (NTM), and the time-consuming culture, which may delay treatment in some critically ill patients (7). The traditional approach in STB diagnosing poses the same limitations (8).

Histopathological diagnosis is of key value in the diagnosis of STB. However, unlike soft tissue lesions such as lymph nodes, STB is a bony lesion, making pathological sectioning more difficult and affecting its diagnostic efficacy (9).

Recent advents in molecular biology have led to the widespread use of molecular diagnostic techniques with improved diagnostic capability for TB (10, 11). XpertMTB/RIF is the most widely used molecular test, and it has proven to be effective in the diagnosis of both PTB and EPTB (12, 13). Likewise, it performs well in term of STB as well (14). In addition to Xpert MTB/RIF, another popular molecular test is the CapitalBio *Mycobacterium* real-time polymerase chain reaction (RT-PCR) assay (CapitalBio test, CapitalBio Technology Inc., Beijing, China), which has shown to be as accurate as Xpert MTB/RIF in diagnosing PTB and certain forms of EPTB (15, 16). The diagnostic accuracy of the CapitalBio test in STB has not been reported in studies. The purpose of this study was to evaluate the diagnostic efficacy of the CapitalBio test in STB. The value of histopathology combined with the CapitalBio test in diagnosing STB was also evaluated, providing more information for clinicians on histopathology and molecular testing.

## Materials and methods

### Study design

We designed a retrospective study at the Zhejiang TB Diagnosis and Treatment Centre, Affiliated Hangzhou Chest Hospital, Zhejiang University School of Medicine to evaluate the diagnostic accuracy of CapitalBio test diagnostic STB and the diagnostic value of CapitalBio test combined with histopathology for STB.

### Participants

Participants in this study were patients with suspected STB admitted to our center between January 2019 and January 2022. Patients with suspected STB were defined as those with STB imaging changes, TB-related symptoms, positive TB-related immunological tests, or combined PTB or other EPTB. Patient clinical information was retrieved and recorded through the electronic medical record system.

### Outcomes

The primary endpoints for this study are generally test accuracy outcomes measured in terms of sensitivity, specificity, positive predictive value (PPV), negative predictive value (NPV), and area under the curve (AUC). After evaluating the outcomes of each test, we further evaluated the outcomes of the two tests employed in combination. A positive result for any single test is considered positive for the combined test.

### Target conditions

Participants were patients with both a CapitalBio test and histopathology performed simultaneously on spinal-related material. Patients with missing test results from relevant tests, or incomplete clinical information were excluded. We signed informed consent from patients or their guardians. This study complies with the Declaration of Helsinki. The Human Research Ethics Committee of the Affiliated Hangzhou Chest Hospital, Zhejiang University School of Medicine, approved the protocol of this study.

### Reference standards

**Figure 1:**
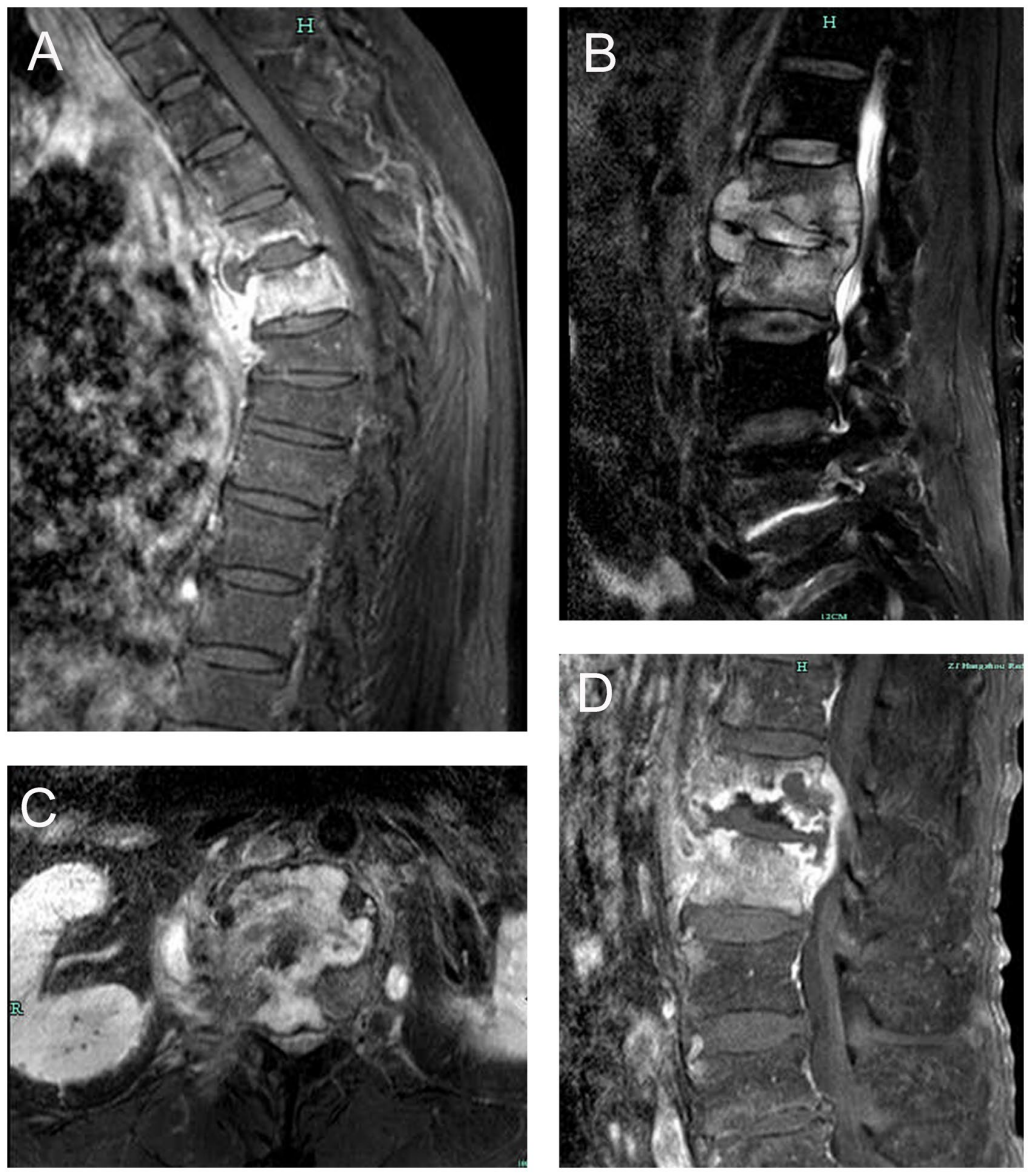
Magnetic resonance imaging changes of spinal tuberculosis. **(A)** thoracic tuberculosis leading to destruction of vertebral body and prevertebral abscess. **(B)** lumbar tuberculosis leading to destruction of vertebral body, intervertebral disk and prevertebral abscess. **(C)** lumbar tuberculosis leading to destruction of lumbar vertebral body. **(D)** lumbar tuberculosis leading to worm-bite like destruction of vertebral body and intervertebral disk.

The composite reference standard served as the diagnostic reference standard for this investigation (CRS). The included patients were segregated into two groups based on the CRS. Patients with typical symptoms of TB poisoning, magnetic resonance imaging of the spine suggestive of TB, positive TB immunoassay (interferon-γ release assays), positive AFB smear or culture in spine samples, positive other TB nucleic acid amplification tests (such as Xpert MTB/RIF), combined PTB or other EPTB, effective anti-TB treatment were diagnosed with STB. Magnetic resonance imaging changes of STB was presented in Figure 1. Patients with negative AFB smear and culture, no evidence of TB infection, clear other causes of spinal lesions, and ineffective anti-TB treatment were diagnosed as non-STB.

### Diagnostic specimen collection and handling

Specimens of pus or tissue from spinal lesions were used in the CapitalBio test, and tissue specimens were used in histopathology. Depending on the severity of the lesion, we obtained the relevant specimen by vertebral puncture or surgery. The fresh specimens were used for traditional *Mycobacterium* testing, CapitalBio, and histopathological testing.

### Histopathology

Spinal tissue specimens fixed in 10% formalin solution were used for histopathology. The pathologist rinsed the fixed tissue specimen and gradually dehydrated it with 80–100% alcohol. The dehydrated specimens were placed in a paraffin solution to make wax blocks. The wax blocks were trimmed and cut into 4–6 μm thick slices. The sections were flattened, placed on slides, and baked at 60°C for 15–30 min to remove the paraffin. Paraffin-removed sections can be used for histopathological examination. The histopathological diagnosis of TB was made when granulomatous inflammation with coagulative necrosis was observed, and a trained pathologist made all diagnoses. In cases of uncertainty, a second expert specialized pathologist was consulted. Histopathology results are usually available within 1 week.

### CapitalBio test

Fresh pus or tissue was used for the CapitalBio test. Pus can be used directly, while tissue specimens need to be cut up, ground, and used. The buffer was added to the initially treated specimen and shaken to obtain a homogenized solution. 1 mL of homogenized specimens was added to 2 mL of specimen preparation solution to obtain liquefied specimens. Sediment was obtained by centrifugation of 1 mL of the liquefied specimen. Nucleic acids were extracted from the sediment. Take 2 μL of the extracted nucleic acid and proceed to the next step according to the product instructions. The presence of MTB is detected by amplifying and detecting the MTB IS6110 multicopy elements using a fluorescence quantification RT-PCR instrument (SLAN-96S Real-Time PCR System ZEESAN Xiamen CN). Test results can usually be reported within 3 h (17).

### Data processing and statistical analysis

Use SPSS 24.0 to calculate the values in the diagnostic cross-tabulation: true positive (TP), false positive (FP), false negative (FN), and true negative (TN). These values were then used in MedCalc Statistical v15.2.2 (MedCalc Software bvba, Ostend, Belgium; http://www.medcalc.org) to calculate the sensitivity, specificity, positive predictive value (PPV), negative predictive value (NPV), and area under the curve (AUC) with 95% confidence interval (CI) of the relevant tests. MedCalc Statistical was also used to plot the receiver operating characteristic curve (ROC). McNemar’s test was used to compare the differences between the two sets of paired data. Chi-square test or Fisher’s exact test was used to compare the difference between the proportions. Z test for comparing the difference between the AUCs. Venn diagram was drawn via an online tool^1^ (18). A *p* value of less than 0.05 was considered a statistically significant difference between the two groups compared.

## Results

We screened 234 individuals for STB and eliminate 12 owing to insufficient data; hence, 222 suspected STB were included in the final study. Of the 222 patients, 125 (56.3%) were male. The ages ranged from 18 to 89 years, with an average age of 61.7 ± 15.0. A total of 46 cases combined with PTB. The information about patient demographics and clinical characteristics was shown in Table 1. All patients tested negative for human immunodeficiency virus. Only 1 AFB smear was positive in all patients, and 86 MTB cultures were positive. Of all patients, 77 were histopathology positive, and 93 were positive for the CapitalBio test.

**Table 1:** The demographic and clinical characteristics of the suspected patients.

Characteristics	All (222)	STB (121)	Non-STB (101)
Age year (Mean ± SD)	61.7 ± 15.0	58.9 ± 16.9	65.1 ± 11.6
Male (*n*, %)	125 (56.3)	67 (55.4)	58 (57.4)
Lesion site (*n*, %)			
Cervical spine	4 (1.8)	2 (1.7)	2 (2.0)
Thoracic spine	103 (46.4)	67 (55.4)	36 (35.6)
Lumbar spine	111 (50.0)	50 (41.3)	61 (60.4)
Sacral spine	4 (1.8)	2 (1.7)	2 (2.0)
Comorbidity (*n*, %)			
Diabetes	36 (16.2)	17 (14.0)	19 (18.8)
Hypertension	66 (29.7)	27 (22.3)	39 (38.6)
Pulmonary tuberculosis	46 (20.7)	42 (34.7)	4 (4.0)
CAHD	12 (5.4)	6 (5.0)	6 (5.9)
Laboratory examinations			
WBC (×10^9^/L, mean ± SD)	5.6 ± 1.5	5.5 ± 1.8	6.2 ± 2.5
RBC (×10^12^/L, mean ± SD)	4.4 ± 0.5	4.5 ± 0.6	3.7 ± 0.6
Platelets (×10^12^/L, mean ± SD)	198.3 ± 40.7	209.7 ± 38.3	261.5 ± 105.5
ESR (mm/h, mean ± SD)	36.9 ± 25.0	35.1 ± 20.3	61.6 ± 28.6

STB, spinal tuberculosis; SD, standard deviation; CAHD, coronary atherosclerotic heart disease; WBC, White blood cells; RBC, Red blood cells; ESR, erythrocyte sedimentation rate.

**Figure 2:**
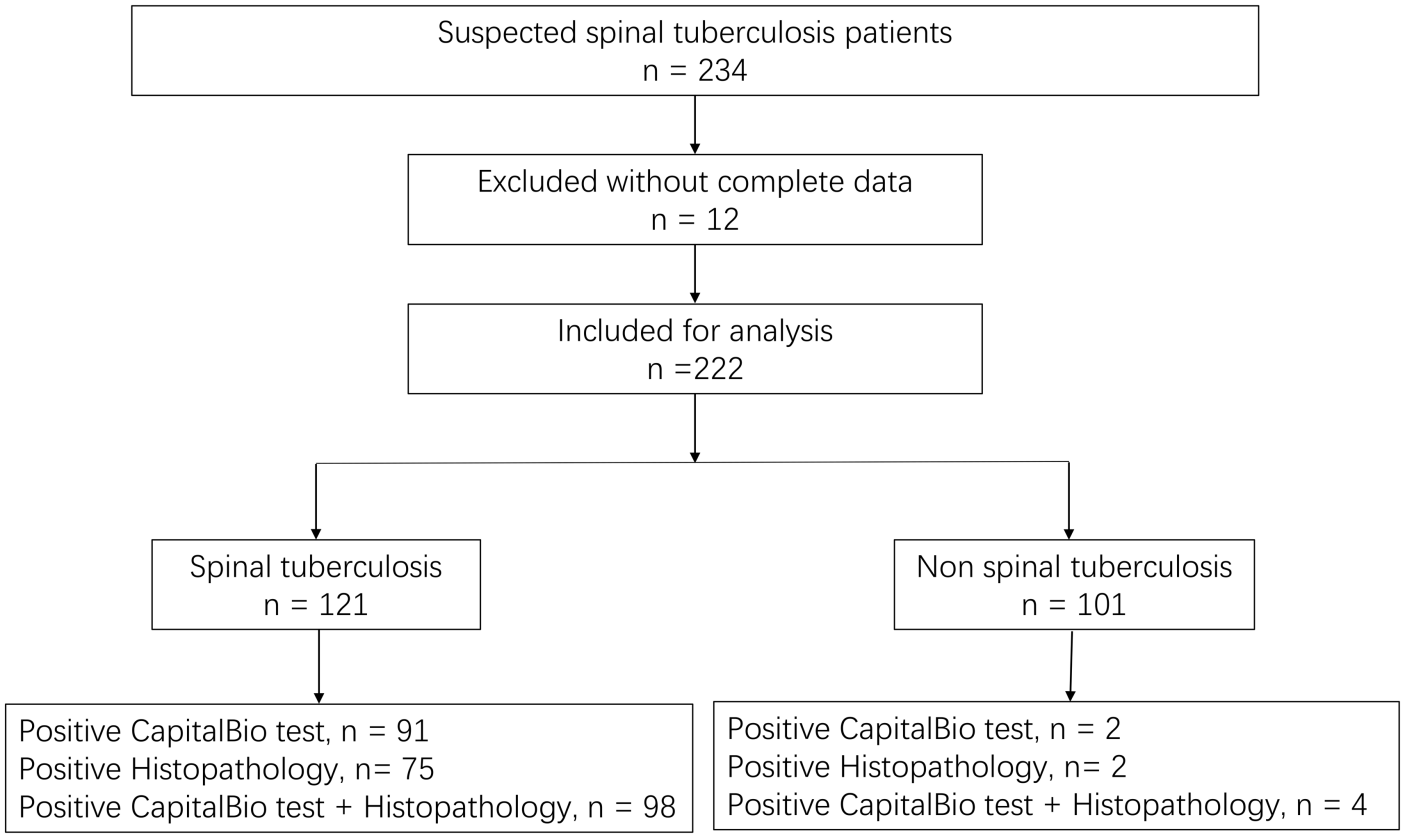
Diagnostic classification of patients included in the study.

**Figure 3:**
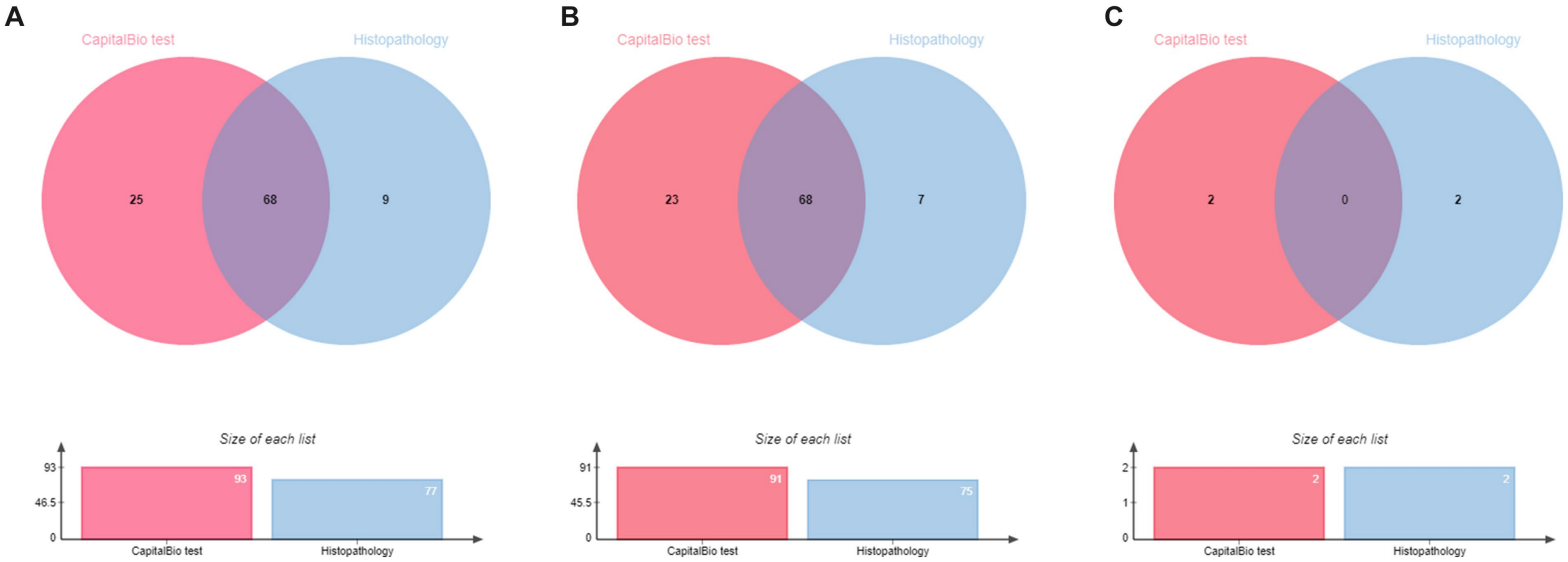
Venn diagram of the distribution of positive patient results in different classifications. **(A)** All patients. **(B)** Spinal tuberculosis patients. **(C)** Non spinal tuberculosis patients.

Based on the diagnostic reference criteria of this study, 121 cases were finally diagnosed as STB, and the remaining 101 cases were diagnosed as non-STB (Figure 2). The distribution and the overlap of positive histopathology and CapitalBio test among all patients are shown in Figure 3A. Sixty-eight cases were positive for histopathology and the CapitalBio test, nine were positive for histopathology only, and 25 were positive for the CapitalBio test only. The distribution of histopathology positive and CapitalBio test positive in the STB group was shown in Figure 3B, and the distribution of the non-STB group was shown in Figure 3C. Eleven of the non-STB cases were eventually diagnosed as NTM infections and 24 cases were diagnosed as spinal tumors.

### Diagnostic accuracy of histopathology, CapitalBio test, and histopathology combined with CapitalBio test

**Figure 4:**
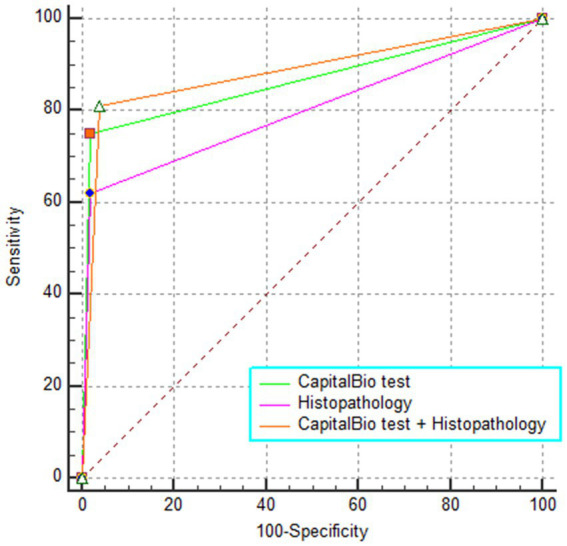
The receiver operating characteristic curve for different tests.

The sensitivity, specificity, PPV, NPV, and AUC of histopathology, CapitalBio test, and histopathology combined with CapitalBio test for STB were reported in Table 2, and the ROCs are exhibited in Figure 4. The overall accuracy of histopathology and CapitalBio test for the diagnosis of STB was satisfactory.

**Table 2:** Accuracy of histopathology, CapitalBio test, and histopathology combined with CapitalBio test for the diagnosis of spinal tuberculosis against a composite reference standard.

Test	Sensitivity (%)	Specificity (%)	PPV (%)	NPV (%)	AUC
Histopathology	62.0 (52.7–70.7)	98.0 (93.0–99.8)	97.4 (90.9–99.7)	68.3 (60.0–75.8)	0.80 (0.74–0.85)
CapitalBio test	75.2 (66.5–82.6)	98.0 (93.0–99.8)	97.9 (92.5–99.7)	76.7 (68.5–83.7)	0.87 (0.81–0.91)
CapitalBio test + Histopathology	81.0 (72.9–87.6)	96.0 (90.2–98.9)	96.1 (90.3–98.9)	80.8 (72.6–87.4)	0.89 (0.84–0.92)

PPV, positive predictive value; NPV, negative predictive value; AUC, area under the curve.

### Comparison of the diagnostic accuracy of these tests

CapitalBio test has far higher sensitivity than histopathology (*p* < 0.05; Table 3), but the overall diagnostic accuracy did not reach statistical differences (*p* > 0.05 for inter-AUC comparisons; Table 3). The combined test did not significantly improve the diagnostic accuracy for STB compared to the CapitalBio test (*p* > 0.05; Table 3). However, compared with histopathology, the combined test significantly improves the diagnostic efficacy of STB (*p* = 0.001; Table 3).

**Table 3:** Comparison of the diagnostic efficiency between these tests for spinal tuberculosis.

Test	Sensitivity (*p*-value)	Specificity (*p*-value)	PPV (*p*-value)	NPV (*p*-value)	AUC (*p*-value)
CapitalBio test vs. Histopathology	0.027	1.000	0.848	0.118	0.064
CapitalBio test vs. CapitalBio test + Histopathology	0.277	0.407	0.474	0.431	0.317
Histopathology vs. CapitalBio test + Histopathology	0.001	0.407	0.626	0.020	0.001

PPV, positive predictive value; NPV, negative predictive value; AUC, area under the curve.

## Discussion

The spine plays a key part in the body, making the consequences of STB potentially catastrophic. The distinct nature of spinal lesions makes it is relatively difficult to obtain spinal specimens. Effective diagnostic approaches are needed to improve STB diagnostic efficiency and reduce the risk of repeated invasive operations causing further damage to patients. Our study suggested that AFB smear was of limited help in the diagnosis of STB. Out of 121 STB patients, only one sample was detected positive with AFB smear, suggesting that AFB smear was almost ineffective in STB diagnosis. The diagnostic ability of current findings was low compared to previous studies (19), probably because the vast majority of specimens included in this study were spinal tissues, which are difficult to process for bony tissues, where the MTB load may also be low. Therefore the number of positive AFB smear was very low. This research revealed 86 positive cultures, which was higher than the number of positive histopathology cases, suggesting that culture was still valid for diagnosing STB. Although MTB culture can effectively identify TB infection, however, it is time-consuming, which is not conducive to rapid diagnosis and effective treatment, and can easily lead to misdiagnosis and adverse outcomes if the culture results are the only factor to rely upon (20). On the other hand, culture can distinguish NTM infections, with 11 cases identified in this study, which is impossible with an AFB smear (21).

Histopathology is the first test to be performed after obtaining specimens for all solid lesions with invasive procedures, and other relevant tests can be considered under the premise of ensuring histopathological testing. Histopathology is a very important part of STB diagnosis. In this study, there were 77 patients with histopathology suggestive of TB infection. Its sensitivity, specificity, PPV, NPV, and AUC for STB were 62.0, 98.0, 97.4, 68.3%, and 0.80, respectively. The accuracy of histopathology in diagnosing STB is relatively good. Still, it is lower compared to culture, probably because bony tissue can make pathological sectioning difficult and because the typical TB changes in bone tissue are not as pronounced as in soft tissue. Histopathology is not a substitute for microbiological evidence, and other diseases may have TB-like pathological alterations (22). In this study, granulomatous inflammation and necrosis were observed on histopathology in two patients with non-STB, but the final culture diagnosis was NTM infection. Histopathology is the gold standard for confirming the diagnosis of spinal tumors and cannot be replaced by other tests. 24 patients in this study were finally diagnosed with spinal tumors by histopathology. These findings suggest that histopathology lacks specificity for STB and that further diagnostic accuracy is best improved by combining it with other relevant tests.

Molecular tests are becoming increasingly valuable in STB (23). This study evaluated the value of the CapitalBio test for the diagnosis of STB. The results suggested that in STB, the sensitivity, specificity, PPV, NPV, and AUC of the CapitalBio test were 75.2, 98.0, 97.9, 76.7%, and 0.87, respectively. No other studies evaluating the validity of the CapitalBio test in STB diagnosis were identified. Still, these results were similar to other molecular tests based on MTB DNA detection (23). Since the CapitalBio test can identify an MTB gene fragment, and amplification by PCR improves the detection of this fragment, its diagnosis accuracy was better than that of histopathology, but the difference between the two did not reach statistical significance. It was suggested that both tests were valid for the diagnosis of STB, but the CapitalBio test can be seen as microbiological evidence. In molecular assays, when fragment amplification through PCR is used, this might result in false positives. In this study, among non-STB patients, two patients had weakly positive results for the CapitalBio test, which may be related to amplification in the test. These two patients were negative for other TB-related tests and cured without anti-TB treatment. The cost of a single CapitalBio test is very low, only one ninth of that of Xpert MTB/RIF, and the test can detect both MTB and NTM, which is very helpful for the diagnosis of possible NTM infection, a task that would have been impossible with Xpert MTB/RIF (17). In this investigation, 11 patients had positive results for NTM on the CapitalBio test, and NTM was cultivated from their samples. The CapitalBio test may be more useful and acceptable for distinguishing between tuberculosis and nontuberculous mycobacterium infections.

Single diagnostic tests may have some shortcomings that combined tests may avoid. We further evaluated the diagnostic accuracy of the histopathology combined with the CapitalBio test. It was found that histopathology combined with the CapitalBio test improved the diagnostic accuracy of STB, and the difference between them was statistically significant when compared to histopathology, whereas it failed to achieve statistical significance upon comparison with the CapitalBio test. In the STB group, 68 patients were positive for histopathology and the CapitalBio test. In other words, the CapitalBio test provided further microbiological evidence in 90.7% (68/75) of patients with positive histopathology, which was of great diagnostic benefit and can greatly reduce misdiagnosis due to similar histopathological changes caused by other diseases. Histopathology combined with the CapitalBio test maximized the diagnostic efficacy, and when sufficient specimens are available, it recommended to perform additional CapitalBio test based on histopathology for STB.

This study experienced some challenges in the designing and experimental stages. Firstly, this study was conducted in a regional TB center and was also a retrospective study, and patient selection may have been biased. Secondly, this study used CRS as the diagnostic reference standard, which includes a variety of evaluation criteria that may vary from patient to patient, leading to a biased result. Thirdly, this study was conducted in an area with a high prevalence of TB, and its findings may not be applicable in other areas.

## Conclusion

Both histopathology and the CapitalBio test exhibited high accuracy in STB diagnosis. The difference between the two was not statistically significant. Histopathology combined with the CapitalBio test might give the best efficacy in STB diagnosis, and it recommended to perform additional CapitalBio test based on histopathology for STB, if possible.

## Data availability statement

The original contributions presented in the study are included in the article/supplementary material, further inquiries can be directed to the corresponding authors.

## Ethics statement

The studies involving human participants were reviewed and approved by The Human Research Ethics Committee of Affiliated Hangzhou Chest Hospital, Zhejiang University School of Medicine. The patients/participants provided their written informed consent to participate in this study.

## Author contributions

JZ and DZ: conceptualization. DZ, LY, and CB: methodology. LY: software, formal analysis, visualization, funding acquisition, and writing – original draft preparation. LY, CB, and JZ: validation. CB: investigation. JZ: resources. LY and CB: data curation. DZ: writing – review & editing and project administration. JZ: supervision. All authors contributed to the article and approved the submitted version.

## Funding

LY, 2022ZB266, Administration of Traditional Chinese Medicine of Zhejiang Province. The funder does not have a role in study design, data collection and analysis, decision to publish, preparation, execution, interpretation, or writing of the manuscript.

## Conflict of interest

The authors declare that the research was conducted in the absence of any commercial or financial relationships that could be construed as a potential conflict of interest.

## Publisher’s note

All claims expressed in this article are solely those of the authors and do not necessarily represent those of their affiliated organizations, or those of the publisher, the editors and the reviewers. Any product that may be evaluated in this article, or claim that may be made by its manufacturer, is not guaranteed or endorsed by the publisher.

## Footnotes

^1^ https://bioinfogp.cnb.csic.es/tools/venny/index.html
